# Supplementary material for: A Review of the Nutritional Approach and the Role of Dietary Components in Children with Autism Spectrum Disorders in Light of the Latest Scientific Research
Source: Nutrients. 2023 Nov 21;15(23):4852. doi: 10.3390/nu15234852 (PMC10708497; doi:10.3390/nu15234852)
Supplement: Supplementary file 1 [file nutrients-15-04852-s001.zip › nutrients-2664328-supplementary.pdf]

Table S1. Studies analyzing the effect of selected dietary treatments on autistic individuals

Intervention	Duration	Subjects	Treatment-related measures	Main results	Authors and Study Design
Comprehensive				Treatment/Nontreatment group	
Nutritional and					
Dietary Intervention	12	67 children	Blood draw and urine		Adams et al.[52]
	months	and adults	collection		
Day 0:		3-58 years			Randomized,
Vitamin/Mineral			BUN	Small decrease	controlled, single-
supplementation			RBC	Significantly decrease	blind treatment study
begins.			EPA, DHA	Large increases	
Day 30: Essential Fatty			AA, LA, DGLA	Small decreases	
Acid supplementation			CRP	No difference	
begins.			Vitamin B_2, B_5, folik asit,	Large, significant increases	
Day 60: Epsom salt			CoQ10H2		
baths begin.			Selenium, chromium	Significant increase	
Day 90: Carnitine			Homocysteine	Significant decrease	
Supplementation			L-carnitine	Significant increase	
begins.					
Day 180: Digestive			BMI	No significant change	
Enzyme					
supplementation			Scales		
begins.			ADOS	No significant change	
Day 210: Healthy,			RIAS	Non-verbal IQ test improvement	
casein-free, gluten-			CARS	Improvement	
free, soy-free diet			SAS-Pro	Improvement	
begins.			VABS2	Improvement (communication, social, and daily	
Day 365: Final				living domains)	
assessment			ATEC	Greater improvement	
			PDD-PI	Greater improvement	

			SRS<br>6-GSI<br>SSP<br>ABC<br>PGI-2	Greater improvement<br>Improvement (constipation, diarrhea, stool smell,)<br>Greater improvement<br>Greater improvement<br>Greater improvement	
GFCF diet	12 months	Only for children with high urinary peptide levels (20 out of 30)<br>6-12 years	Biological matrixes (blood, urine)	Very remarkable decrease in casomorphin and gliadomorphin concentrations<br><br>After 6 months: A large decrease in essential elements (iron, calcium, zinc, and magnesium) concentrations for the majority of children<br>**-Modified and fortified diet**<br>Remarkable improvement in the nutritional status	Hafid and Ahami. [53]<br><br>Trial
			CARS	Significant decrease in 12 subjects	
A modified ketogenic gluten-free diet (daily total net carbohydrates 20–25 g, protein RDA requirement) with supplemental MCT (20% of energy needs)	3 months	15 children<br>2-17 years	ADOS-2<br><br>CARS-2	Significantly improved core autism features assessed from the ADOS-2<br><br>Significant improvements in CARS-2 (imitation, body use, and fear or nervousness)	Lee et al. [54]<br><br>Open-label, observer-blinded clinical trial

GFCF diet<br>Time 0: Prior to any dietary intervention.<br>Time 1: Upon finishing the first dietary intervention (after the normal diet in group A and after the GFCF diet in group B).<br>Time 2: Upon finishing the second dietary intervention (after the GFCF diet in group A and after the normal diet in group B).	3 months	28 children<br>3-16 years	Urinary Concentrations of Beta-Casomorphin	No statistically significant differences in the three-time points.	González-Domenech et al. [55]<br>Crossover clinical trial
			ABC	No significant difference from one time point to the next in both groups	
			ATEC Scale	Both groups showed statistically significant differences among the three time points assessed; a paired subanalysis of time points within each group showed significant differences only in group B. In group A, the scores decreased after the normal diet and after the GFCF diet.	
			ERC-III	No statistical significance in group A among the three time points assessed. Statistically significant differences were found in group B among the three time points assessed and when paired analysis was performed.<br>In general, group B had more behavioral changes than group A	

A diet including gluten and casein (normal diet) for 6 months and a GFCF diet for another 6 months.<br><br>Grup A: Normal diet (6 months)<br>GFCF diet (6 months)<br><br>Grup B: GFCF diet (6 months)<br>Normal diet (6 months)	12 months	37 children 2-18 years	Urinary Concentrations of Beta-Casomorphin	After GFCF non-significant decrease	González-Domenech et al. [56]<br><br>Crossover clinical trial
			**Hemogram and Biochemistry**<br>Calcium, vitamin D, ferritin, folic acid, IGF-1 and hematocrit	No significant difference	
			Questionnaire for gastrointestinal disorders	No changes in the 27 participants presenting with a history of gastrointestinal or eating disorders.	
			Weight-height Monitoring	No significant difference	
			ABC	No significant differences	
			ERC-III	Group A showed significant decreases (after the GFCF)<br>When both groups were analyzed together non-significant decrease in the scores after the GFCF	
			ATEC Scale	No significant differences	
GFD	6 months	66 children (33 children who had been on a GFD for at least 8	ADOS-2	Treatment/Nontreatment group<br>No significant differences between groups at baseline and at the 6-month follow-up<br><br>(RRB domain score) Significant improvements (In both groups, between baseline and the 6-month follow-up revealed)	Piwowarczyk et al. [57]<br><br>A randomized, controlled, single-blinded trial

weeks before enrollment) 36-69 months	SCQ, ASRS, VABS-2, Leiter Scale	No significant differences between groups) (SCQ and ASRS) Significant improvements (In both groups, between baseline and the 6-month follow-up revealed)
	BMI	No significant differences
	ROME-III Gastrointestinal Symptoms	(Abdominal pain, constipation) No significant differences

AA= Arachidonic acid, ABC = The Aberrant Behavior Checklist Scale, ADOS = Autism Diagnostic Observation Schedule, ASRS= Autism Spectrum Rating Scale, ASD = autism spectrum disorder, ATEC = Autism Treatment Evaluation Test questionnaire, BMI=Body Mass Index, BUN=Blood Urea Nitrogen, CARS = Childhood Autism Rating Scale, CRP= C-Reactive Protein, DHA=Docosahexaenoic acid, DGLA= dihomo-y-linolenic acid, EPA= Eicosapentaenoic acid, ERC = Evaluation Resumé du Comportement, in French = The Behavioral Summarized Evaluation, GFCF = gluten free casein free diet, GFD = gluten free diet, IGF-1= Insulin-like Growth Factor 1, LA= Linoleic acid, PDDBI= Pervasive Developmental Disorders Behavior Inventory, PGI2= Parent Global Impressions-2, RBC=Red Blood Cell, RIAS= Reynolds Intellectual Assessment Scales, RRB= Restricted and Repetitive Behaviors, SASPro= Severity of Autism Scale, SCQ = Social Communication Questionnaire, SSP= Short Sensory Profile, SRS = Social Responsiveness Scale, VABS = Vineland Adaptive Behavior Scale, 6-GSI= 6-item Gastrointestinal Severity Index

Table S2. The effects of prebiotic and probiotic supplementation on the health status of individuals with ASD.

Intervention	Duration	Subjects	Treatment-related measures	Main results	Authors and Study Design
Supplementation of probiotics<br>Daily dose 5 g<br>Each gram contains 100 × 10^6 colony forming units of three probiotic strains (Lactobacillus acidophilus, Lactobacillus rhamnosus, and Bifidobacteria longum)	3 months	30 autistic children<br>5-9 ages	Stool samples (PCR)	Significant increases in the levels of both Bifidobacterium and Lactobacillus	Shaaban et al. [151]<br>Prospective, open-label study
			BMI	Significant reduction	
			ATEC	Significant improvements	
			6-GSI	Significant improvements	
Supplementation of prebiotic<br>Bimuno® galactooligosaccharide (B-GOS®)<br>Prebiotic	6 weeks	30 autistic children<br>4-11 ages	Quantitatively evaluation	A general trend of reduction in GI problems (not significant)	Grimaldi et al. [150]<br>Intervention study
			Qualitative analysis	Sleep habits improvoment (23% of participants)	
Exclusion diet (like GFCF)			Anxiety and ASD-related behaviour questionnaires	A significant improvement in social behaviour scores (in exclusion group)	
Un-restricted diet			ATEC	Consistent reduction over time in anti-sociability score (in exclusion group)	

			FISH analysis	Increase in the number of Bifidobacterium spp<br>Not a significant difference between treatments<br><br>Bacterial populations positively associated with B-GOS supplementation on the unrestricted diet.	
			Gut microbiota composition	Significant alterations (in the unrestricted group)	
			Urine spectra profiles	Ethanol, DMG and SCFAs (butyrate, valerate) were positively correlated with B-GOS® intake	
			Fecal samples	Metabolic shifts<br>Lower levels of amino acids (isoleucine, leucine, valine, alanine, glutamine) and lactate (compared to placebo)	
Supplementation of probiotic/colostrum<br><br>BCP (bovine colostrum product) as a source of prebiotic oligosaccharides<br><br>Combination group: BCP+*Bifidobacterium infantis* in<br><br>BCP only group<br><br>with a	12 weeks<br><br>5 weeks of probiotic+ prebiotic supplementation, followed by a two-week washout period<br>5 weeks only prebiotic supplementation	11 children with ASD 2–11 years (with a history of frequent gastrointestinal symptoms including chronic constipation, diarrhea, and/or	ABC	A significant reduction in certain aberrant behaviors, irritability, stereotypy, hyperactivity, total scores and a trend toward a significant reduction in lethargy (for BCP only)<br>A significant reduction only in lethargy (for the combination group)<br>Significant improvement in stereotypy in the BCP-only group	Sanctuary et al. [49]<br>A randomized, double-blind, controlled trial
			RBS-R<br>ABAS-II	No differences in repetitive behaviors<br>No differences in adaptive behaviors	
			GIH/<br>QPGS-RIII	Some improvement in GI symptoms (87.5% BCP only; 100.0% combination group)	

The colostrum powder dose 0.15 g/lb body weight per day		irritable bowel syndrome (IBS))	Consumption novel foods	Increased appetite and consumption of novel foods (43.0% for BCP only; 14.0% for combination group)	
BCP was 5.1–10.8 g per day depend on the child's weight			Bristol Stool Scale	A significant increase in the percentage of normal consistency stools (in combination group)<br>A trend toward a significant decrease in hard stool for week 1 versus week 5 (for the BCP only)<br>A reduction in the frequency of diarrhea (for BCP only)	
			Microbiota analysis	No global changes in the gut microbiota	
			Microbial community state analysis	No effect or an inconsistent effect on enterotype (for both groups).	
			Blood collection and cellular assays	In stimulated cells, the frequency of CD4+/IL-13+ T cells was significantly lower after combination treatment<br>A significant reduction in the frequency of D8+/TNF-α+ T cells with the BCP-only treatment	
			Serum, urinary and fecal metabolomics	No global changes in fecal, urinary or serum metabolite profiles	
Supplementation of probiotics<br>De Simone Formulation (DSF)<br>Vivomixx® in EU, Visbiome® in USA<br>2 packets/day in the first month and 1	6 months	85 autistic children 18-72 months	**ADOS-CSS**<br>SA<br>RRB<br>VABS II<br>GMDS-ER<br>Total GSI<br>Total 6-GSI	From baseline (T_0) to T_2<br>No significant differences in the probiotic vs the placebo group (secondary outcomes)	Santocchi et al. [154]<br>Double-blind randomized, placebo-controlled trial

packet/day in the following 5 months. Each packet contained 450 billions of eight probiotic strains:	SCQ<br>SP<br>RBS-R<br>CBCL 1,5-5<br>PSI	
Streptococcus thermophilus, Bifidobacterium	Total ADOS-CSS	A significant decrease (for NGI probiotic group compared to that in the placebo group)
breve, Bifidobacterium longum, Bifidobacterium infantis,	ADOS-CSS (Social affect, Total)	A significant decrease (for NGI probiotic group compared to that in the placebo group)
Lactobacillus acidophilus, Lactobacillus plantarum,	GI semptoms	Statistically significant effects (Total GSI , Total 6-GSI, stool smell, flatulence mean scores) in the GI group
Lactobacillus para-casei, Lactobacillus delbrueckii subsp. Bulgaricus	Adaptive functioning (Receptive Skills, Domestic Skills and Coping Skills VABS-II subscales)	Statistically significant effects in the GI group
Probiotics group and control group (T_0:Baseline time T_2: at 6 months time)	A normalization of Sensory Profile scores in the Multisensory Processing subscale	Significantly higher the GI group
Subgroups of children with or without Gastrointestinal	BMI	Not significant
	**Blood samples**	From baseline to T2 in all the subjects

Symptoms (GI/NGI) (according to Gastrointestinal Severity Index)				Not significant	
			IL-6	Not significant	
			Leptin	Not significant	
			TNF-a	Not significant	
			PAI-1		
			**Fecal samples**	From baseline to T2 in all the subjects	
			Calprotectin	Not significant	
Probiotic supplementation<br><br>The feeding product (whey powder without casein and some minced cooked yellow vegetables in adequate ratios fortified with the studied probiotic strains (Bifdobacterium spp. and Lactobacillus spp.)) (10^8 /g)<br>10 g of the nutritional supplement once a day	3 months	40 children with ASD 2-5 years	Fecal samples	A signifcant increase in the colony counts of both Bifdobacterium spp. and Lactobacillus spp.<br>Highly signifcant value in the case of Bifidobacterium spp. (p-value 0.001) and a signifcant increase in the case of Lactobacillus spp	Meguid et al. [124]
			Weight and BMI	A highly significant decrease	
			ASQP	An improvement	
			Sleep disturbances	An improvement	
			**CARS (Total score)**	Highly significant improvement	
			The relation to people, Body use, Visual response, Verbal communication, Intellectual response	Highly significant improvement	
			Imitation, Object use, adaptation to change, fear	Significant improvement	

		or nervousness, nonverbal communication	
		QPGS-RIII	Some improvement in GI symptoms (abdominal pain, including stomach aches and other abdominal discomforts in the upper abdomen), decreases in the number of poops and changes in stool consistency to be a little soft

ABAS-II: Adaptive Behavior Assessment System–Second Edition; ABC: Aberrant Behavior Checklist; ADIR: Autism Diagnostic Interview-Revised; ADOS-CSS: Autism Diagnostic Observation Schedule – Calibrated Severity Score; ASQP: Anxiety Screening Questionnaire; ATEC: Autism Treatment Evaluation Checklist; BMI: Body Mass Index; CARS: Childhood Autism Rating Scale; CBCL: Child Behavior Check List; DMG: Dimethylglycine; FISH: Fluorescence in situ hybridisation; GFCF: Gluten Free Casein Free; GI: Gastrointestinal; GIH: Gastrointestinal History; GMDS-ER: Griffiths Mental Development Scales-Extended Revised; Il-6: Interleukin-6; PAI: Plasminogen Activator Inhibitor; PCR: Polymerase Chain Reaction; PSI: Parenting Stress Index; QPGS-RIII: Questionnaire on Pediatric Gastrointestinal Symptoms-Rome III Version; RBS-R: Repetitive Behavior Scale-Revised; RRB: Restricted Repetitive Behaviours; SA: Social-Affect; SCFAs: Short-chain fatty acids; SCQ: Social Communication Questionnaire; SP: Sensory Profile; TNF-a: Tumor Necrosis Factor-alpha; VABS-II: Vineland Adaptive Behavior Scales-Second Edition; 6-GSI: GI Severity Index.
